# Potential for Bacteriophage Endolysins to Supplement or Replace Antibiotics in Food Production and Clinical Care

**DOI:** 10.3390/antibiotics7010017

**Published:** 2018-02-27

**Authors:** Michael J. Love, Dinesh Bhandari, Renwick C. J. Dobson, Craig Billington

**Affiliations:** 1 Biomolecular Interaction Centre and School of Biological Sciences, University of Canterbury, Christchurch 8041, New Zealand; michael.love@pg.canterbury.ac.nz (M.J.L.); dinesh.bhandari@esr.cri.nz (D.B.); renwick.dobson@canterbury.ac.nz (R.C.J.D.); 2 Institute of Environmental Science and Research, Christchurch 8041, New Zealand; 3 Department of Biochemistry and Molecular Biology, University of Melbourne, Melbourne 3052, Australia

**Keywords:** endolysin, antibiotics, antimicrobial resistance, one health, protein engineering

## Abstract

There is growing concern about the emergence of bacterial strains showing resistance to all classes of antibiotics commonly used in human medicine. Despite the broad range of available antibiotics, bacterial resistance has been identified for every antimicrobial drug developed to date. Alarmingly, there is also an increasing prevalence of multidrug-resistant bacterial strains, rendering some patients effectively untreatable. Therefore, there is an urgent need to develop alternatives to conventional antibiotics for use in the treatment of both humans and food-producing animals. Bacteriophage-encoded lytic enzymes (endolysins), which degrade the cell wall of the bacterial host to release progeny virions, are potential alternatives to antibiotics. Preliminary studies show that endolysins can disrupt the cell wall when applied exogenously, though this has so far proven more effective in Gram-positive bacteria compared with Gram-negative bacteria. Their potential for development is furthered by the prospect of bioengineering, and aided by the modular domain structure of many endolysins, which separates the binding and catalytic activities into distinct subunits. These subunits can be rearranged to create novel, chimeric enzymes with optimized functionality. Furthermore, there is evidence that the development of resistance to these enzymes may be more difficult compared with conventional antibiotics due to their targeting of highly conserved bonds.

## 1. Introduction

In 2014, the World Health Organization (WHO) calculated the global prevalence of seven antibiotic-resistant bacteria of international concern, and noted very high rates of resistance (up to 84% of all isolates for methicillin, 81% for third-generation cephalosporins, 49% for fluoroquinolones, and 60% for penicillin) in all WHO regions [1]. In response to this unprecedented crisis, in late 2016 the United Nations General Assembly called upon the WHO, the Food and Agriculture Organization of the United Nations, and the World Organisation for Animal Health to develop a global development and stewardship framework [2]. This request recognized that there was a need to co-ordinate action against antimicrobial resistance in humans, agriculture, animals, and the environment by using a One Health [3] approach. One of the key recommendations in the draft framework was to develop new antimicrobial agents for use in these key sectors.

Here, we discuss the potential of cell wall lysis proteins (endolysins) derived from bacteriophages for use as a new class of antimicrobial agents, and evaluate whether they could replace, or supplement, some of the conventional antibiotics used to treat animals and humans, and perhaps even find use in food production and environmental decontamination processes. 

**Figure 1:**
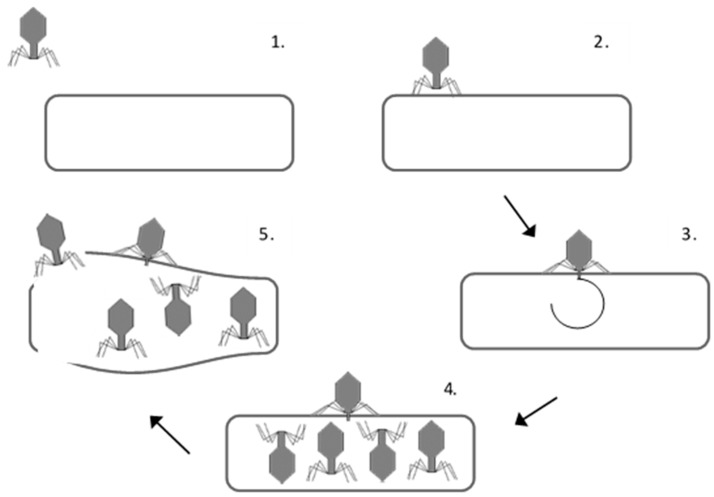
Life cycle of a virulent tailed phage (not to scale). (1) The phage collides with the bacterial cell; (2) the phage binds to cell receptors; (3) the phage is irreversibly bound and injects nucleic acid into the cell via the tail tube, where it is transcribed and translated; (4) many progeny phages are produced within intact cells; (5) endolysins degrade the host bacterial cell wall, which loses its structural integrity and ruptures due to the osmotic pressure, releasing the progeny phages.

Endolysins are enzymes encoded by bacteriophages (phage; obligate viruses of bacteria) which lyse the host bacterial cell. Endolysins degrade the main structural component of the cell wall (peptidoglycan) at the conclusion of the replicative cycle to release newly assembled progeny phage [4] (Figure 1). Recombinantly expressed endolysins display similarly effective lytic abilities to their native counterparts when applied exogenously to susceptible bacteria [5]. This feature underpins the application of endolysins in medicine, food and agriculture.

Endolysins are predominantly more effective against Gram-positive bacteria than Gram-negative bacteria when applied in this way. The outer membrane of Gram-negative bacteria presents a physical protective barrier against the activity of endolysins [6]. Therefore, endolysin research has mainly focused on Gram-positive bacteria. However, recent work on outer membrane permeabilizers (chemicals and engineered peptides) should increase the prospects of endolysins for treating Gram-negative bacteria.

**Figure 2:**
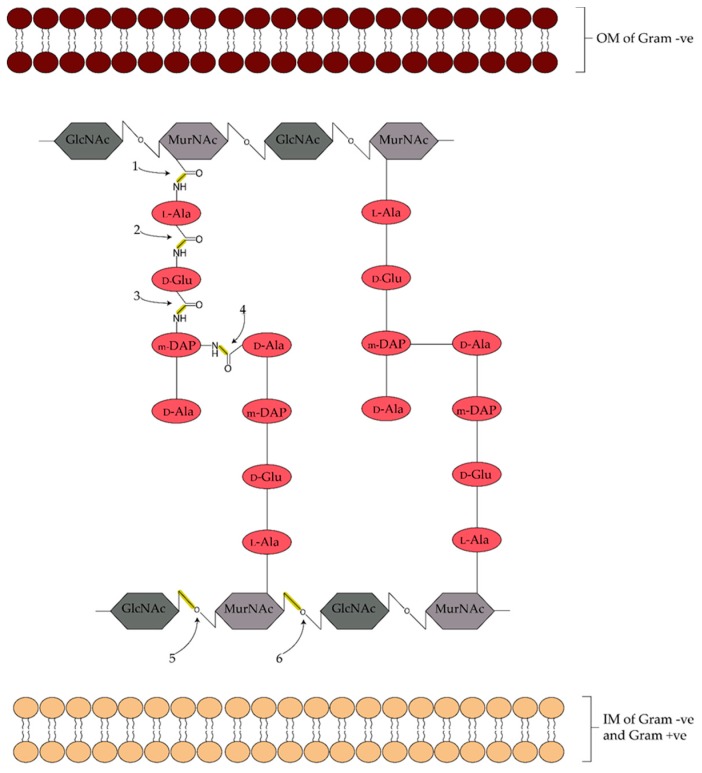
Diagram of the typical cell wall and peptidoglycan structure of bacteria, including the endolysin cleavage sites. The peptidoglycan is composed of repeating sugar units, *N*-acetylglucosamine (GlcNAc) and *N*-acetylmuramic acid (MurNAc), which are cross-linked via an interpeptide bridge between the *meso*-diaminopimelic acid (m-DAP) and d-alanine (d-Ala) residues of adjacent tetrapeptide chains. The chains also contain _L_-alanine (l-Ala) and _D_-glutamic acid (d-Glu). Gram-negative bacteria contain an outer membrane (OM) structure not present in Gram-positive bacteria. Both contain an inner membrane (IM) structure. The cleaved bonds and major classifications of endolysin are indicated: (1) *N*-acetylmuramoyl-l-alanine amidase; (2–4) various endopeptidases; (5) N-acetyl-β-d-glucosaminidase; (6) N-acetyl-β-d-muramidase (lysozyme).

Numerous types of endolysins have been described, and are typically categorized by the structural bonds in the peptidoglycan that are cleaved by the enzyme [7] (Figure 2). The two alternating glycosidic bonds between the amino sugar moieties, *N*-acetylglucosamine and *N*-acetylmuramic acid (MurNAc), are targeted by different endolysin classes. The *N*-acetylmuramoyl-β-1,4-*N*-acetylglucosamine bond is cleaved by lytic transglycosylases and *N*-acetyl-β-d-muramidases, which are commonly known as lysozymes, while the *N*-acetylglucosaminyl-β-1,4-*N*-acetylmuramine bond is hydrolysed by *N*-acetyl-glucosaminyl-β-d-glucosaminidases. The cleavage of the amide linkage between MurNAc and l-alanine is catalyzed by *N*-acetylmuramoyl-l-alanine amidases. There are different endopeptidases depending on the chemical structure of the peptidoglycan, which is dependent on species and growth conditions. Generally, endopeptidases hydrolyze the peptide bonds between the amino acids that form the cross-linking peptide stems [8,9,10].

A number of promising endolysins have been isolated from phage for application as antimicrobial agents, as described in this review and others [5,6,7,8,9]. However, the bioengineering of modified or novel endolysins also holds promise for the future development of effective tools to kill or detect bacteria. The prospects for engineering are facilitated by the enzymes’ structures. The domain structure of endolysins can be modular (Gram-positive bacteria and some Gram-negative species) or globular (most Gram-negative bacteria), with most bioengineering strategies exploiting the modular endolysins. These enzymes usually comprise two distinct domains: an N-terminal enzymatically-active domain and a C-terminal cell wall-binding domain, connected by short, flexible linker regions. N-terminal enzymatically-active domains are responsible for catalyzing the breakdown of specific peptidoglycan bonds, while the C-terminal cell wall-binding domains recognize and bind non-covalently to substrate within the cell wall, resulting in the specificity of the lytic enzymes for the target host [5,8]. In addition, the C-terminal cell wall-binding domain is often required to maintain full lytic activity [11,12,13]. Interestingly, truncation or deletion of the C-terminal cell wall-binding domain can also result in equal or increased lytic activity [14,15,16]. In contrast, globular endolysins only contain catalytic domains.

The modular endolysin arrangement can be exploited for bioengineering, as the different domains can be shuffled within the protein, or domains from different endolysins can be combined to generate new enzymes [17,18]. Directed mutagenesis is also an effective strategy, as different amino acids may support improved lytic or binding properties [5,8,17,18]. The seemingly limitless possible permutations of endolysin modular arrangements allow for the development of new enzymes with specific functions or features. Some potential bioengineering strategies and examples of endolysin constructs are shown in Table 1.

Both bioengineered and phage-isolated endolysins are promising alternatives to antibiotics. Their specificity allows them to target specific bacterial pathogens without affecting the microflora [11], or alternatively, target a larger spectrum for broader efficacy [27]. At the moment, developed resistance to the activity of the endolysins has not been widely reported, meaning these enzymes could be a long-term solution to antibiotics [28,29,30]. Endolysins may also have potential as diagnostic tools for bacterial identification [31]. In the following sections, we provide an overview of native and chimeric endolysins with potential therapeutic applications. 

## 2. Endolysins as Human Therapeutics

As the efficacy of antibiotics decreases, once easily-treated bacterial infections will become potentially fatal. This will also have secondary effects in clinical care, such as changing risk-benefit considerations for invasive surgeries. Phage-encoded lytic enzymes have the potential to fulfil the need for novel antibacterial therapeutic agents for use in humans. This new class of antimicrobials has been recognized by the United States of America in the National Action Plan for Combating Antibiotic-resistant Bacteria [32], which identified the use of “phage-derived lysins to kill specific bacteria while preserving the microbiota” as a key strategy to reduce the development of antimicrobial resistance.

Methicillin-resistant *Staphylococcus aureus* (MRSA) is a significant public health concern, causing a range of skin and respiratory infections, as well as food-borne illnesses that are not easily treatable with currently available antibiotics [33]. O’Flaherty et al. [27] treated a human-derived MRSA strain with *Lactococcus lactis* cell lysate containing recombinantly overexpressed endolysin LysK, and observed a 99% reduction in colony-forming units at 1 h post-exposure. However, the researchers had difficulties obtaining soluble protein, which would hinder future applications of LysK, a difficulty that was also encountered in subsequent studies [34,35]. A stability study was conducted on LysK, as medical application requires a stable enzyme [34]. LysK was stabilized in the presence of low molecular weight polyols such as sucrose and glycerol, for example, stability increased 100-fold at 30 °C, and LysK retained 100% activity after storage up to 1 month at room temperature. This stability, under simple condition changes, is useful for developing treatment strategies [34]. LysK contains two catalytic domains: a cysteine- and histidine-dependent amidohydrolase/peptidase (CHAP) domain, and an *N*-acetylmuramoyl-l-alanine amidase domain. In an attempt to overcome the solubility issue, Horgan et al. [19] generated a single-domain truncated LysK mutant, designated CHAPk, containing only the CHAP domain. Soluble CHAPk was easier to obtain than full-length LysK, and displayed at least a two-fold increase in lytic activity against both heat-killed and live staphylococcal cells in vitro. Subsequent studies demonstrated that CHAPk was also effective in vivo, and that the loss of the C-terminal cell wall-binding domain, which directs specificity, resulted in activity against a broader range of targets compared with full-length CHAPk [36,37].

Jun et al. [38] compared LysK with SAL-1, an endolysin that differs by three residues. They also produced six derivatives of SAL-1 containing mutations in each of the three residues to investigate the impact of each mutation. SAL-1 displayed cell-wall hydrolytic activity approximately two-fold greater than that of LysK. The mutation of residue 114 from glutamatic acid in LysK to glutamine in SAL-1 had the largest impact on activity. This residue is located inside the catalytic CHAP domain, and the structurally minor sequence change corresponded to enhanced activity. The combination of such enhanced activity with the identification and mechanistic characterization of key residues of different enzymes is important for rational design and engineering of new endolysins with optimized activity [38]. Compared with LysK, SAL-1 had increased catalytic activity, and high yields of soluble protein were easier to obtain; therefore, SAL-1 may be a more promising antibiotic alternative than LysK [39]. The therapeutic application of SAL-1 is currently being trialed by iNtRON Biotechnology in the form of SAL200, an endolysin-based candidate drug for the treatment of *S. aureus*. A preclinical safety study of SAL200 observed no toxicity in rodent intravenous single- and repeat-dose studies [40]. A repeat-dose experiment was also performed in dogs, with each dog receiving four doses of 0, 0.5, 12.5 and 25 mg/kg in 1-week intervals over four weeks. After ten days, short-lived (i.e., lasting only 30 minutes to 1 hour) and mild clinical signs were observed including, vomiting, subdued behavior and irregular respiration. The transient response of the dogs to SAL200 administration was linked to complement system activation that resulted from antibody production [40]. A follow-up study in monkeys investigated the impact of single-dose escalation (up to 80 mg/kg) or 5-day multiple-dose (up to 40 mg/kg/day) administration of SAL200, with no adverse effects observed [41] SAL200 was further evaluated in a human single dose-escalating (up to 10 mg/kg) study. This study was the first in-human clinical study of a intravenously administered endolysin-based drug [42]. The volunteers had a reasonable tolerability to SAL200, with no significant adverse effects and most of the adverse effects were mild and self-limited. Although an increased concentration of antibodies was observed, the antibody concentration of participants administered 3.0 mg/kg was greater than that of those administered 10 mg/kg, with large variation within the different cohorts. The immunogenicity of SAL200 should, therefore, be a focus of future studies in order to better develop treatment regimes [42]. A phase II clinical study is now being conducted on patients with persistent *S. aureus* bacteremia, with results expected in 2018. Overall, the current evaluations shows a promising future for not just SAL200, but also for the development of other endolysin-based drug treatments.

Biofilm formation in clinical environments and on medical devices can have significant medical implications, as biofilms can harbor pathogenic and multidrug-resistant bacteria. Microorganisms within biofilms are protected by extracellular polymeric substances (EPS), which are a source of environmental contamination when partially dislodged. EPS can contain polysaccharides, proteins, phospholipids, teichoic acids, nucleic acids, and polymers, and protect the biofilm inhabitants by concentrating nutrients, preventing access of biocides, sequestering metals and toxins, and preventing desiccation [43]. Linden et al. [44] found that recombinantly-expressed PlyGRCS (from the phage GRCS) effectively lysed *S. aureus* in a biofilm, as well as in stationary phase. PlyGRCS contains a single enzymatically-active domain that can cleave two different bonds in peptidoglycan. This bifunctional domain could be highly useful in developing endolysins with effective lytic activity. 

Rashel et al. [45] found that a dose of the phage ϕMR11-derived lysin MV-L rescued mice from fatal levels of MRSA exposure. In addition, MV-L in combination with vancomycin killed vancomycin-resistant strains. MV-L was specific for *S. aureus* and *Staphylococcus simulans*, with no lytic activity observed against other staphylococcal strains or bacterial species, including *Staphylococcus epidermidis* and *Escherichia coli*. Although excessive exposure to MV-L induced the production of antibodies, no adverse effects on the mice or impact on the efficacy of MV-L was observed.

Daniel et al. [20] demonstrated the potential of bioengineering to generate enzymes with novel and specific lytic activity against MRSA. The endolysin ClyS was constructed from the enzymatically active domain of a *S. aureus* Twort phage lysin fused with the cell wall-binding domain of phiNM3. Mice were exposed to MRSA strains that were resistant to the antibiotic oxacillin. A dose of ClyS increased survival rates to 88%, compared with the 0% survival rate for untreated mice. Treatment of infected mice with a sub-therapeutic concentration of ClyS in combination with oxacillin increased survival rates when compared with each treatment alone. This synergistic relationship with antibiotics may have widespread potential, and reinitiate the use of historical antibiotics that have been discontinued due to resistance concerns.

Schuch et al. [46] further showed this synergistic potential with the lysin CF-301. Mice with staphylococcal-induced bacteremia had a survival time of less than 24 h without treatment. Following individual treatments with CF-301 and daptomycin at 4 h post-inoculation, survival rates after 72 h were measured at 13% and 23%, respectively. Combination therapy yielded a survival rate of 73%. The study further confirmed the efficacy of co-therapy in 16 individual experiments including the antibiotics oxacillin and vancomycin. The immunogenicity of CF-301 was briefly evaluated in vitro; rabbit antisera raised against CF-301 did not inhibit the activity of CF-301 [46]. Despite the in vitro results, the immunogenic nature of CF-301 needs to be studied in a range of model organisms in vivo, because there may be clinically relevant adverse effects. CF-301 also has anti-biofilm activity [47], and clinical phase I trials are now underway to evaluate CF-301 as an alternative to traditional antibiotics, with an expected study completion in late 2018. 

Thermal injury patients are usually also immunocompromised, meaning they are more susceptible to bacterial infection, including drug-resistant *S. aureus* strains [48]. Chopra et al. [49] investigated the use of endolysin MR-10 alone and in combination with minocycline to treat burn wound infections in mice. The control mice, inoculated with *S. aureus* and receiving no medical treatment, had a 100% mortality rate within 5 days. Individually, MR-10 (50 µg/ml) and minocycline (50 mg/kg) both resulted in a survival rate of 35% at 5 days post-inoculation, but 100% mortality was observed by day 7. In contrast, 100% survival was observed following treatment with a combination of the therapeutic agents at the same concentrations. These findings further support the future use of endolysins in medicine, especially in co-therapy with existing antibiotics. 

Staphefekt is an endolysin bioengineered to selectively target *S. aureus* strains, including MRSA. It is currently available as a component of gels and creams for over-the-counter treatment of infections [50,51]. Its specificity for *S. aureus* is an important feature in the treatment of skin infections, as it prevents the disturbance of commensal bacteria, which can cause further health complications [52]. Evidence for the efficacy of Staphefekt is limited; there are few available publications describing the rationale of engineering or the in vitro properties and structure of the endolysin. However, a recent report by Totté et al. [51] demonstrated the efficacy of Staphefekt in treating three different patients with recurrent *S. aureus*-related dermatoses that had previously been unsuccessfully treated with antibiotics. Despite the limited published evidence, these brief findings suggest a promising future for Staphefekt as a long-term alternative to antibiotics.

*Enterococcus faecalis* is the third most common cause of life-threatening nosocomial infections [53], and is intrinsically resistant to antibiotics [54]. Although vancomycin is considered a drug of last resort, a growing number of vancomycin-resistant *E. faecalis* strains have been isolated. [55,56]. An endolysin isolated from phage ϕ1, PlyV12, kills a variety of *E. faecalis* strains in vitro, including vancomycin-resistant isolates. PlyV12 also showed a broad spectrum of lethality against a variety of streptococcal and staphylococcal strains, highlighting it as a promising candidate in antibacterial medicine [57]. Son et al. [58] also identified a novel phage, EFAP-1, which was not significantly similar to any previously identified phages. The endolysin of EFAP-1, EFAL-1, exhibited lytic activity against 24 different strains of bacteria, including vancomycin-resistant *E. faecalis* strains and four streptococcal strains, whereas the phage itself only showed activity against *E. faecalis.* There is a lack of in vivo studies of *E. faecalis* endolysins. Such studies are important because *E. faecalis* is also a commensal bacterium found in the human gut, and therefore the potential impact of endolysins on the gut microbiota needs to be understood [52]. Zhang et al. [59] isolated phage IME-EF1 and its endolysin from hospital sewage, and investigated their ability to rescue mice from lethal challenge with *E. faecalis*. Individually, both the phage and the endolysin reduced the bacterial count in the blood of infected animals. However, a 200-µg dose of the endolysin at 30 min post-bacterial inoculation supported a higher survival rate (80%) than that observed following phage treatment alone (60%) [59].

The endolysin LysEF-P10, derived from *E. faecalis* phage EF-P10, has also been studied in a mouse model. A single dose of just 5 µg was enough to protect mice from vancomycin-resistant *E. faecalis* infection, suggesting promising protective efficacy of LysEF-P10 against vancomycin-resistant *E. faecalis* strains. Furthermore, when the mice were subjected to a large dose of 5 mg, no side effects were observed. The administration of EF-P10 stimulated specific antibody production, however, there was no impact on the bactericidal activity of the enzyme. Treatment with EF-P10 did not negatively affect the gut microbiota, owing to the specificity of the lysin. Although *E. faecalis* in the normal gut microbiota may have been targeted, no significant health impact on the mice was observed [60]. Proença et al. [23] constructed a bacteriolysin-like enzyme to target *E. faecalis*. The construct, EC300, was created from the fusion of the peptidase domain from a virion-associated lysin and the cell wall-binding domain of Lys170, both found in phage F170/08. EC300 inhibited the growth of *E. faecalis* in bacterial culture media, whereas the parental endolysin Lys170 showed limited inhibition. The enhanced lytic and killing ability of EC300 highlights the potential for engineered endolysins compared with wild-type enzymes.

Streptococcal infections are associated with a range of clinical manifestations, including strep throat, pneumonia, skin infections, and meningitis [61,62]. Drug resistant streptococcal strains are also increasing in prevalence. Loeffler et al. [28] examined the potential of the endolysin Pal to kill *S. pneumoniae* that had colonized the nasopharynx of mice. Pal was effective against 15 different strains of pneumococci, including some drug-resistant strains, reducing *S. pneumoniae* to undetectable levels within 5 h of treatment. The same research group [63] then examined the antibacterial ability of a previously described lytic enzyme, Cpl-1 [64]. Rabbit antiserum was raised against Cpl-1 and the impact on bacterial lysis measured. Only a small amount of inhibition was measured, showing the antibodies had little effect on the enzymatic activity [63]. In the same study, these findings were corroborated in vivo. Mice that were subjected to several doses of Cpl-1 tested positive for IgG. These immunized mice along with naïve mice were challenged with *S. pneumoniae*. Comparatively, no significant difference was measured with regards to the reduction of bacteria numbers by the enzyme [64]. Mice intravenously infected with *S. pneumoniae* and treated only with buffer had a median survival time of 30.75 h, with no mice surviving at 72 h post-infection. Mice treated with Cpl-1 at 5 and 10 h post-infection had a median survival time of 60 h, although after 96 h, only one mouse survived. Although the potential for complete eradication of the bacteria was shown, the dosage used in the experiments was not high enough, meaning that all animals eventually succumbed to infection. Therefore, for greater efficacy of Cpl-1, a higher dose would be required [63].

Subsequent studies demonstrated the effectiveness of Cpl-1 combination therapy with lytic enzyme Pal, or with antibiotics [65,66,67]. In vivo mouse model studies demonstrated these Cpl-1 treatments were effective in the treatment of pneumococcal diseases such as sepsis [68], endocarditis [69], meningitis [70], and pneumonia [71,72]. Despite its demonstrable antibacterial activity, a key limitation of Cpl-1 is its half-life in blood of only 20.5 min in mice [63,69]. Resch et al. [72] introduced specific cysteine residues into Cpl-1 to promote disulfide bond formation and subsequent dimerization. Dimerization is required for full activity of LytA, a pneumococcal autolysin [73], with which Cpl-1 shares extensive sequence similarity. Dimerized Cpl-1 displayed a two-fold increase in antimicrobial activity and had a nearly ten-fold decrease in plasma clearance, resulting in an increased half-life. These enhanced properties not only increase the prospects for Cpl-1 application, but also highlight the potential for enhanced activity through structural changes and engineering.

The exogenous treatment of Gram-negative bacteria with endolysins has been limited because of the presence of the outer membrane, which prevents access to the peptidoglycan layer [74]. Overcoming this protective layer is a major obstacle in developing endolysin-based treatments. Most studies have focused on nosocomial pathogens *Acinetobacter baumannii* and *Pseudomonas aeruginosa*, both of which are Gram-negative and capable of forming biofilms. Multidrug-resistant strains of both pathogens are also being increasingly isolated [75,76,77,78]. Some endolysins can intrinsically permeate the outer membrane [79,80,81]. Lai et al. [79] recombinantly expressed LysAB2 from ϕAB2 and applied it to *A. baumannii*. The C-terminus of LysAB2 contains an amphipathic α-helix that interacts with the negatively charged elements of the outer membrane, facilitating the formation of a transmembrane pore [82]. This allows the N-terminus catalytic domain to interact with the peptidoglycan layer and lyse the cell, achieving antibacterial activity. Lood et al. [80] identified and screened 21 different endolysins for sequence diversity and *A. baumannii*-killing activity. The endolysins displayed varying degrees of antibacterial activity, with the lysin PlyF307 exhibiting the greatest activity. The C-terminus of PlyF307 contains a highly positively charged region, which may interact with the outer membrane. PlyF307 successfully killed both planktonic cells and, more importantly, those within biofilms, providing an advantage over antibiotics. PlyF307 also functioned under physiological conditions, rescuing mice treated with lethal doses of *A. baumannii*. There remains a library of lysins for structural and biochemical characterization from this research.

Walmagh et al. [81] showed that OBPgp279, from the phage OBP, can permeate the outer membrane of *P. aeruginosa*. It does not appear to contain an amphipathic helix, and thus the mechanism of permeabilization is unclear. OBPgp279 may therefore contain novel structural elements that could lend themselves to the engineering of new endolysins to target Gram-negative bacteria [81]. The lysin LysPA26 from phage JD010 has antibacterial activity against both planktonic and biofilm-contained *P. aeruginosa* cells, as well as other Gram-negative species such as *E. coli* and *Klebsiella pneumoniae.* However, LysPA26 was ineffective against Gram-positive species, including *S. aureus*. This specificity may allow for selective targeting of Gram-negative species in medical treatments [83].

Strategies employing endolysins in conjunction with antibiotics or outer membrane-permeabilizing agents have been explored. Thummeepak et al. [84] investigated the use of LysABP-01 in combination with colistin for treatment of hospital-isolated strains of *A. baumannii*. Although LysABP-01 alone prevented growth, elevated levels of antibacterial activity were observed in combination with colistin. Additionally, the minimum inhibitory concentrations of LysABP-01 and colistin were reduced 32-fold and by up to eight-fold, respectively, when used in combination, compared with individual application. Endolysin EL188, from phage EL, combined with EDTA reduced *P. aeruginosa* cell counts by up to four log units, whereas EL188 on its own exhibited no antibacterial activity [85]. However, the use of EDTA would be restricted to topical applications because of its ability to inhibit blood coagulation [86].

Artilysin bioengineering has also shown promise in targeting Gram-negative bacteria. Artilysins are created through the fusion of outer membrane-permeabilizing peptides, which interfere with the stabilizing forces within the outer membrane, with endolysins. These fusion proteins allow the uptake of an endolysin across the outer membrane, providing access to the peptidoglycan layer [87]. Initially, two endolysins, OBPgp279 and PVP-SE1g-146, fused with one of seven different peptides were investigated for their antibacterial activity. Although the endolysins exhibited limited antibacterial activity on their own, fusion with polycationic nonapeptide correlated with up to a 2.6 log reduction of *P. aeruginosa* in only 30 min. Moderate antibacterial activity against *A. baumannii*, *E. coli*, and *Salmonella* Typhimurium was also observed. The mode of action was examined using time-lapse microscopy, confirming that the artilysins were passing through the outer membrane and degrading the peptidoglycan [24].

Briers et al. [25] developed Art-175, composed of the antimicrobial peptide (AMP) sheep myeloid 29-amino acid peptide (SMAP-29) fused with endolysin KZ144, to target *P. aeruginosa*. AMPs are involved in the innate immune response, and can move across the outer membrane [88]. Art-175 reduced the *P. aeruginosa* cell count by up to 4 log units compared with untreated controls, and continuous exposure to Art-175 to exert a selection pressure did not elicit the development of resistance. On its own, SMAP-29 is cytotoxic to mammalian cells [89]; however, Art-175 exhibited little toxicity in L-292 mouse connective tissue. As a result of these findings, Peng et al. [26] developed AMPs based on the amino acid sequence of LysAB2. The synthesized AMPs killed *A. baumannii* cells by permeating the outer membrane in vitro. Treatment with the AMPs also increased the survival rate of mice infected with a lethal dose of *A. baumannii* by 60%. This research highlights a novel method of bioengineering endolysins for use as antimicrobials.

## 3. Endolysins as Veterinary Treatments

In response to widespread concern regarding the overuse of antibiotics in food-producing animals, many major food suppliers are now committed to phasing out prophylactic antibiotic use and the tighter control of therapeutic treatments. This presents obvious challenges for the animal-husbandry industry, which may be overcome by the use of endolysins. Companion and working animals can also be susceptible to recalcitrant microbial infections, including those caused by multidrug-resistant microorganisms, and so may also benefit from endolysin treatment.

*Clostridium perfringens* is a leading cause of necrotic enteritis and sub-clinical disease in poultry, and can lead to significant economic losses [90]. Swift et al. [22] constructed recombinant endolysin PlyGVE2CpCWB, which shows enhanced thermostability, an important feature for surviving feed heat treatments. The recombinant endolysin contains an amidase domain from an endolysin derived from a thermophilic phage fused with the cell wall-recognition domain from *C. perfringens*-specific phage endolysin PlyCP26F, which is not resistant to high temperatures. PlyGVE2CpCWB inactivated *C. perfringens* in both liquid and solid media at temperatures up to 50 °C, and so may be a promising antimicrobial feed treatment for controlling necrotic enteritis in poultry. This thermostable construct demonstrates the potential for other thermophilic bacteriophage endolysins to be utilized in bioengineering. A different approach to *C. perfringens* control was taken by Gervasi et al. [91,92], whereby an amidase endolysin (CP25L) was cloned and expressed in a *Lactobacillus johnsonii* strain isolated from poultry. In co-cultures, reductions of up to 2.6 log_10_ CFU·ml^−1^ *C. perfringens* were noted; however, the reduction was inconsistent between experiments, and the effect declined significantly over time. This reduced activity was attributed to a loss in stability of the endolysin in culture, and a reduction in the viability of *L. johnsonii.* Other researchers [93,94] have also performed detailed analyses, including X-ray crystallography, on another *C. perfringens* endolysin, Psm, which may have applications in poultry. Psm is an *N*-acetylmuramidase endolysin with wide activity against *C. perfringens* strains.

Another economically significant disease in animal husbandry is bovine mastitis, which is caused by a variety of bacteria, of which staphylococci and streptococci account for 75% of cases [95]. In studies aimed at treating bovine mastitis caused by *S. aureus*, Schmelcher et al. [21] demonstrated that fusion of an endopeptidase endolysin domain from a *Streptococcus* lambda phage (SA2) with either lysostaphin (SA2-E-Lyso-SH3b) or staphylococcal phage endolysin LysK (SA2-E-LysK-SH3b) could inhibit staphylococci in a murine mammary mastitis model. The extended lytic spectrum targeting multiple genera would be particularly useful for efficiently treating bovine mastitis [96]. Infusion of 25 µg of SA2-E-Lyso-SH3b or SA2-E-LysK-SH3b into the mammary glands reduced *S. aureus* counts by 0.63 and 0.81 log_10_ CFU·mg^−1^, respectively. Additional testing of SA2-E-LysK-SH3b and lysostaphin in combination (12.5 µg/gland) revealed a 3.36 log_10_ CFU·mg^−1^ reduction in the concentration of *S. aureus* compared with the control [21]. Further work by the same group [97], determined the potential of the lambda SA2 and phage B30 (a CHAP endopeptidase) endolysins in combination as a therapeutic treatment of *Streptococcus*-induced mastitis, again in a murine mastitis model. The best results obtained by the study were reductions of 1.5 log_10_ CFU·mg^−1^ against *Streptococcus uberis* (SA2), 4.6 log_10_ CFU·mg^−1^ against *Streptococcus agalactiae* (B30), and 2.2 log_10_ CFU·mg^−1^ against *Streptococcus dysgalactiae* (SA2). More recently, purified endolysin Trx-SA1, isolated from *S. aureus* phage IME-SA1, was used to treat naturally infected cow udders [98]. Udder quarters received an intramammary infusion of 20 mg of Trx-SA1 once per day, and qualitative reductions in somatic cell counts and *S. aureus* numbers were noted over the three-day regime.

Anthrax, a potentially fatal zoonotic disease affecting a wide variety of species, is a threat to wild and farmed animals as well as humans, especially as a biological weapon [99,100]. Schuch et al. [29] reported the usefulness of PlyG lysin, isolated from the γ-phage of *Bacillus anthracis*, in killing vegetative cells and germinating spores of *B. anthracis* and streptomycin-resistant *Bacillus cereus* RSVF1. The researchers screened an expression library of cloned γ-phage DNA sequences and identified a 702-bp open reading frame (ORF) encoding a protein with homology to an amidase-type endolysin. When PlyG was injected intraperitoneally (50 U in 0.5 mL) into mice infected with 6 log_10_ CFU RSVF1, a notable therapeutic effect was observed, with 68.4% (13/19) of mice showing full recovery. Furthermore, the survival time of the remaining mice was prolonged to 21 h post-infection.

Equine strangles is a highly contagious disease of horses caused by *Streptococcus equi*. The disease progresses as an inflammation of the upper respiratory tract, and leads to abscess formation in the retropharyngeal lymph node [101]. Strangles is a significant economic threat to the horse racing industry, where many high value animals are typically housed in close proximity. Hoopes et al. [102] used PlyC, an unusual multimeric amidase-type endolysin [17], as a disinfectant against *S. equi* and reported it to be 1000 times more active on a per weight basis than the widely used disinfectant Virkon-S. PlyC was effective against >20 clinical isolates of *S. equi*, including both *S. equi* subsp. *equi* and *S. equi* subsp. *zooepidemicus*, demonstrating its sterilizing ability against an eight log CFU·ml^−1^ culture of *S. equi* within 30 min of exposure.

The clinical efficacy of a muramidase as a veterinary treatment for companion animals was demonstrated in a trial by Junjappa et al. [103], where they successfully treated 17 dogs suffering from pyoderma (bacterial skin lesions) caused by MRSA. The skin lesions were treated with a hydrogel containing a chimeric endolysin composed of the cell wall-targeting domain (SH3b) of lysostaphin and the phage K ORF56 muralytic domain [104]. Another important zoonotic pathogen, *Streptococcus suis*, has been linked to arthritis, meningitis, septicemia, and endocarditis in pigs, as well as in humans who have come into contact with infected animals or their byproducts. Wang et al. [105] isolated a phage from *S. suis* (SMP), and then expressed the endolysin LySMP in *E. coli* BL21. The resultant product, following chromatography and treatment with β-mercaptoethanol, killing 15 out of 17 clinical *S. suis* serotype 2 isolates from diseased pigs in China, and had demonstrated activity against *S. suis* serotype 7 and 9 strains, *S. equi* subsp. *zooepidemicus*, and *S. aureus* [105].

There is growing evidence to suggest that food-producing animals are an important global reservoir of vancomycin-resistant enterococci (VRE) [106,107]. This group of potentially invasive microorganisms is resistant to almost all of the available antibiotic regimens recommended for treatment of Gram-positive bacterial infections. In an attempt to combat VRE in food-producing animals, Yoong et al. [57] cloned the PlyV12-encoding gene from enterococcal phage Φ1 into the *E. coli-Bacillus* shuttle vector pDG148, followed by its expression in *Bacillus megaterium* strain WH320. The resultant product, an amidase-type endolysin, had lytic activity against 14 clinical and laboratory *E. faecalis* and *Enterococcus faecium* strains, including two vancomycin-resistant *E. faecalis* and three vancomycin-resistant *E. faecium* strains, in addition to its host, *E. faecalis* V12. Intriguingly, PlyV12 also had a significant killing effect on pathogenic streptococcal strains, including *Streptococcus pyogenes* (group A streptococcus) and group C streptococci.

Diarrheal outbreaks caused by *Clostridium difficile* have frequently been reported in animals, including cattle, horses, and pigs [108,109]. Treatment of *C. difficile* diarrhea with antibiotics is not recommended as it can further exacerbate the disease condition [110]. In a quest for an alternative approach to treat infections caused by *C. difficile*, Mayer et al. [111] sequenced the genome of a temperate phage from *C. difficile*. They identified endolysin gene *cd27l* and cloned it into vectors pET15b and pUK200 to express the gene product in *E. coli* and *L. lactis*, respectively. The purified endolysin was active against 30 diverse strains of *C. difficile*, including those belonging to the major epidemic ribotype, 027 (B1/NAP1). Unlike antibiotics, the endolysin was selective for *C. difficile*, demonstrating no activity against a range of commensal species from within the gastrointestinal tract, including other clostridia, bifidobacteria, and lactobacilli.

*Paenibacillus larvae* subsp. *larvae* causes American Foulbrood disease in honey bees, which are important insect pollinators of agricultural crops. The disease occurs in honeybee larvae as a result of *P. larvae* spores germinating in the larval midgut and subsequently causing sepsis and death. The use of antibiotics to treat the disease in the USA now requires supervision by a veterinarian, and a withholding period of 4–6 weeks is recommended for honey from treated hives prior to sale (https://www.fda.gov/AnimalVeterinary/ResourcesforYou/AnimalHealthLiteracy/ucm309134.htm). In the European Union, no veterinary medicines containing antibiotics are permitted in beekeeping (http://europroxima.com/european-legislation-regarding-antibiotics-in-honey-an-overview/). For these reasons, endolysins are being investigated as a potential alternative control tool. An amidase endolysin, PlyPl23, has been cloned from a *P. larvae* phage and subsequently expressed [112]. In bee larvae experimentally infected with spores, PlyPl23 effectively decreased the rate of *P. larvae* infection, and no toxic side effects were noted in the larvae. However, the endolysin was not effective until the spores had germinated.

## 4. Endolysins as Food and Environmental Decontaminants

During post-harvest processing, food is vulnerable to cross-contamination from microbial pathogens, which pose a risk to food safety, as well as from microorganisms that can cause quality or shelf-life defects. Effective interventions for foods and the food processing environment are therefore vital to maintain the integrity of the food supply chain. Endolysins have the potential to be key intervention tools for this purpose. 

The use of endolysins to prevent contamination of ready-to-eat foods by the common food and environmental pathogen *Listeria monocytogenes* has been established by groups from around the world [16,113,114,115,116,117]. Zhang et al. [113] cloned an endolysin gene (*lysZ5*) from the genome of *L. monocytogenes* phage FWLLm3 into *E. coli* and tested the sterilization efficacy of the expressed protein (a murine hydrolase) in soya milk contaminated with *L. monocytogenes*. The purified protein had a bactericidal effect on *L. monocytogenes* growing in soya milk, with the pathogen concentration reduced by more than 4 log_10_ CFU·ml^−1^ after 3 h of incubation at 4 °C. Furthermore, the protein displayed a broad host spectrum, lysing lawn cultures of *L. monocytogenes*, *Listeria innocua*, and *Listeria welshimeri*. In a different approach, van Nassau et al. [114] tested the combined effect of previously characterized endolysins (PlyP40, Ply511, or PlyP825) and high hydrostatic pressure on the survival of *L. monocytogenes*. They reported that the combination of treatments had a synergistic effect, capable of reducing viable cell counts of *L. monocytogenes* by up to 5.5 log_10_, compared with 0.3 and 0.2 log_10_ CFU reductions, respectively, when used alone.

Turner et al. [115] and Gaeng et al. [16] demonstrated that the *L. monocytogenes* endolysin gene *ply511* can be cloned and expressed in *Lactobacillus* spp., which have potential as biopreservatives in foods and as a starter culture for fermented milk products. Further to this, Turner et al. [115] combined the cloned A511 phage *ply511* gene with a lysostaphin-encoding *lss* gene from *S. simulans* biovar *staphylolyticus* in-frame with a Sep secretion signal. The resulting construct, Sep-6_His-Ply511, was able to secrete both Ply511 and lysostaphin from *Lactobacillus lactis*, indicating that this recombinant organism could be used for industrial applications as a preservative to prevent contamination of foods with *Staphylococcus* spp. and *L. monocytogenes*.

Staphylococcal food poisoning caused by heat stable enterotoxins produced by *S. aureus* is frequently reported following the consumption of contaminated food and milk products [118]. Chang et al. [119] tested LysSA11, a *S. aureus* phage SA11-derived endolysin, to determine its bactericidal activity in food and on food utensils artificially contaminated with MRSA. Treatment of artificially contaminated ham and pasteurized milk with endolysin for 15 min resulted in 3.1 log_10_ CFU·cm^−3^ and 1.4 log_10_ CFU·ml^−1^ reductions in viable MRSA, respectively, at refrigeration temperature (4 °C), and 3.4 log_10_ CFU·cm^−3^ and 2.0 log_10_ CFU·ml^−1^ reductions, respectively, at room temperature (25 °C). The same group [120] tested the antibacterial potential of LysSA97 in combination with several active compounds derived from essential oils used by the food industry against *S. aureus*. They reported the superior activity of carvacrol in combination with LysSA97, compared with that of the endolysin alone, in food products including beef and milk contaminated with *S. aureus*. When used alone, LysSA97 and carvacrol reduced *S. aureus* concentrations by 0.8 and 1.0 log_10_ CFU·ml^−1^, respectively; however, a synergistic reduction of 4.5 log_10_ CFU·ml^−1^ was observed when the treatments were combined. Similarly, Obeso et al. [121] and Rodriguez-Rubio et al. [122] demonstrated the potential of phage-derived endolysins LysH5 and HydH5 (a hydrolase), respectively, to protect milk products from *S. aureus* contamination. 

*Salmonella* species are the leading cause of bacterial foodborne illness in the USA and many other countries [123]. *Salmonella* disease outbreaks are associated with a wide variety of food products, including red meats, poultry, and produce [124]. Several recombinant endolysins derived from *Salmonella* phages have been characterized [125,126,127]. Interestingly, many of these endolysins have activity outside of the host species of the parental phage, particularly when cell membrane-disrupting chemicals are used in conjunction with the enzymes. Lim et al. [125] expressed the endolysins and spanin proteins from *Salmonella* phage SPN1S and observed activity against both *Salmonella* Typhimurium and *E. coli* isolates in buffer containing EDTA to destabilize the cell membranes. Furthermore, some activity was also noted against typhoidal salmonellae, *Shigella*, *Cronobacter*, *Pseudomonas*, and *Vibrio* species. Oliveira et al. [126] characterized a thermostable *Salmonella* endolysin, Lys68, that displayed better activity at neutral pH and a wide temperature tolerance, maintaining 76.7% of its activity after 2 months at 4°C and partial activity following exposure to 100 °C for 30 min. Thermostability is a useful feature for diverse application, such as in heat treatment of food When Lys68 was tested in combination with citric or malic acid against *S.* Typhimurium LT2, up to 5 log_10_ CFU reductions were achieved, with cells in stationary phase or in biofilms also reduced by up to 1 log_10_ CFU [126]. More recently, Rodriguez-Rubio et al. [127] reported a *Salmonella* phage endolysin, Gp110, that possessed both a novel enzyme structure and N-acetylmuramidase lysis domain, and had unusually high in vitro activity against *Salmonella* and other Gram-negative pathogens [127].

Around the turn of the century, a rare but frequently fatal disease of neonatal infants was first reported to be associated with contamination of powdered infant milk formula with *Enterobacter* (now *Cronobacter*) *sakazakii* [128]. It is now known that several species of *Cronobacter* can cause a variety of diseases, including sepsis and severe meningitis, in neonates, as well as respiratory and urinary tract infections in elderly and immunocompromised individuals. These opportunistic pathogens are now under much scrutiny because of their ability to survive heat, desiccation, and acid stress, which poses a risk of contamination of various milk powders, herbal teas, and other dried products (https://www.cdc.gov/cronobacter/technical.html). Enderson et al. [129,130] expressed and purified a peptidoglycan hydrolase (LysCs4) from *C. sakazakii* that had the highest sequence similarity to a putative lysozyme from the temperate *Cronobacter* phage ES2. The purified lysozyme could degrade the peptidoglycan from both Gram-negative and Gram-positive bacteria belonging to six different genera, and could lyse outer membrane-permeabilized *C. sakazakii*. Similarly, the previously discussed endolysins SPN1S [125] and Lys68 [126] are active against permeabilized *C. sakazakii* cells.

Several groups have investigated the potential of endolysins active against *B. cereus* as biocides and preservatives for use in the food industry [131,132,133]. *B. cereus*, a Gram-positive spore-forming bacterium, is known for its ability to cause food poisoning by producing both an emetic toxin and a diarrheal toxin [131]. Loessner et al. [132] isolated and characterized three endolysins (PlyBa, Ply12, and Ply21) from the *B. cereus* phages Bastille, TP21, and 12826, respectively, and tested their efficacy against a range of Gram-positive and Gram-negative bacteria. They reported that all three endolysins were effective against 24 strains of *B. cereus*, along with several strains of *B. thuringiensis*. Ply12 and Ply21 were found to be *N*-acetylmuramoyl-l-alanine amidases, while PlyBa could not be classified at the time, but is also likely to be an amidase (http://www.uniprot.org/uniprot/P89927). Park et al. [133] isolated a putative endolysin gene from the genome of *B. cereus* phage BPS13, and expressed it in *E. coli*. The purified LysBPS13 protein, an amidase, retained its lytic activity against *B. cereus* ATCC 10876 even after incubation at 100 °C for 30 min, demonstrating its potential as a decontaminant in food processing applications. In contrast, Son et al. [131] proposed *L*-alanoyl-d-glutamate endopeptidase LysB4 as a potential biocontrol agent against *B. cereus* and other pathogenic bacteria. They confirmed the endopeptidase had a broad range of bactericidal activity against Gram-positive bacteria, including *B. cereus*, *B. subtilis*, and *L. monocytogenes*, and also a few Gram-negative bacteria. The endolysin showed optimum lytic activity at pH 8–10 and at 50 °C, making it a suitable candidate for use in the food industry.

In addition to causing diseases in poultry, clostridial species are linked to food spoilage. In the dairy industry, germinated *Clostridium sporogenes* and *Clostridium tyrobutyricum* can contribute to the production of gases and acids that change the structural and sensory qualities of cheeses [134]. Mayer et al. [135] isolated an *N*-acetylmuramoyl-l-alanine amidase, CS74L, from *C. sporogenes* and reported that the purified protein effectively lysed *C. sporogenes* cells when added exogenously. Using the turbidity assay and fresh bacterial cells, the authors also demonstrated that CS74L was active against *C. tyrobutyricum* and *Clostridium acetobutylicum*, making it a candidate biopreservative for use in cheese. The same group also characterized another endolysin isolated from a virulent phage, CPT1l, but this enzyme had a more limited host range [134].

The dairy industry has a long-held interest in utilizing endolysins to control the cheese maturation process. Vasala et al. [136] isolated a muramidase, Mur, from the LL-H phage of *Lactobacillus delbrueckii* subsp. *lactis* that had activity against cell wall preparations of *L. delbrueckii* subsp. *lactis, L. delbrueckii* subsp. *bulgaricus*, *Lactobacillus acidophilus*, *Lactobacillus helveticus*, and *Pediococcus damnosus.* Similarly, Deutsch et al. [137] purified endolysin Mur-LH from a phage infecting the Swiss cheese starter *L. helveticus*. This muramidase had activity against other lactobacilli, *Leuconostoc lactis*, *P. acidilactici*, and, surprisingly, against *B. subtilis*. Kashige et al. [138] isolated an *N*-acetylmuramoyl-l-alanine amidase from phage PL-1, which was originally isolated from an abnormal fermentation of a lactic acid beverage produced using a *Lactobacillus casei* strain. There are also many examples of endolysins isolated from lactococci, including from phages P001, C2 US3, and TUC2009 [136]. A survey of 18 *L. lactis* phage endolysins revealed that muramidases and amidases predominate [139].

In addition to the aforementioned enzymes, several other endolysins with potential to be used against a range of foodborne microorganisms in different food types have been identified, and selected examples of these are illustrated in Table 2.

The growth of biofilms in food processing environments leads to an increased risk of microbial contamination of foods [148]. There are some examples of endolysins being used to disrupt biofilms with relevance to the food industry. Gutierrez et al. [149] investigated the activity of three endolysins (LysH5, CHAP-SH3b, and HydH5-SH3b) against biofilms formed by two *S. aureus* isolates from food. Preformed biofilms were treated with 7 µM of the enzymes, with LysH5 and CHAP-SH3b most effective against strains IPLA1 and Sa9, respectively. In another study, an *N*-acetylmuramoyl-l-alanine amidase was used to disrupt *Listeria* biofilms in vitro, but was found to be most effective when used in combination with a protease [150]. The *Salmonella*-phage endolysin Lys68 also reduced the concentration of cells in a *S.* Typhimurium LT2 biofilm by up to 1.2 log_10_ CFU [128], but this required the presence of either citric or maleic acid to permeabilize the cell membranes.

Phytopathogenic bacteria have a significant global economic cost, and are the cause of multiple food security issues [151]. The use of antibiotics in plant agriculture is controversial because its contribution to the development of antibiotic resistance in human pathogens is undetermined [152] Although its impact may be small, ideally, an alternative strategy to control phytopathogenic bacteria will be developed. As such, the use of endolysins to protect plants from bacterial diseases has been proposed [16]. Widespread implementation of these endolysins will, however, be a significant challenge because of the vast number of crops that would require treatment, and the presence of beneficial soil-borne bacteria. A proposed strategy is the development of transgenic crops that express endolysins, providing protection against the pathogenic bacteria. The potential of this approach has been demonstrated by Düring et al. [153], who produced transgenic potato plants expressing T4 lysozyme. These engineered plants displayed resistance to *Pectobacterium carotovora* (formerly *Erwinia carotovora*) species, which are the cause soft rot [153,154]. Wittmann et al. [155] produced transgenic tomato plants expressing the endolysins from bacteriophage CMP1 in an attempt to prevent *Clavibacter michiganensis* subsp. *michiganensis* infection, the causative agent of bacterial wilt and canker [156]. No symptoms of bacterial infection were observed in the transgenic plants; however, the bacteria were not completely eliminated [155]. A key limitation of this research was that the bacterial infection model may not be representative of natural infection, and therefore the efficacy of these transgenic tomato plants should to be evaluated under more natural conditions. It is also unknown whether *C. michiganensis* subsp. *michiganensis* could acquire resistance to these transgenic plants. However, this is a promising advancement in the development of transgenic plants. As new endolysins are characterized, more opportunities for bioengineering to optimize the activity of the protection mechanism will be possible. 

*Xanthmonas oryzae* pv. *oryzae* causes bacterial leaf blight in rice [157], with a number of antibiotic resistant strains having been isolated [158]. In 2006, Lee et al. [159] identified Lys411 from ΦXo411, which exhibited strong lytic activity against *Xanthmonas*. Additionally, it displayed activity against the multidrug-resistant bacterium *Stenotrophomonas maltophilia* [159], which has growing clinical significance with regards to nosocomial infections and immunocompromised patients [160]. However, no follow-up studies investigating Lys411 have been published, which means the potential of this enzyme for medical or agriculture applications is unknown. 

Attai et al. [161] recently characterized an endolysin from bacteriophages Atu_ph02 and Atu_ph03 for the biocontrol of *Agrobacterium tumefaciens. A. tumefaciens* is a Gram-negative soil-borne bacterium that is the etiologic agent of crown gall disease in a variety of orchard and vineyard crops [162]. Its severity and widespread impact has contributed to it to becoming the subject of many recent studies [163]. The lytic protein displayed interesting properties, with the ability to not only rapidly lyse the cell, but to also block cell division, ensuring potent antimicrobial activity [161]. Therefore, the enzyme is a candidate for biocontrol of *A. tumefaciens*; however, the method of implementation needs to be researched before a viable strategy for crop protection can be developed. The practicalities of implementing these endolysins on a global scale for individual phytobacteria may be a significant challenge, and may contribute to the limited information currently available on the use of endolysins for treatment of plant bacterial diseases. However, the cost to society of plant bacterial disease as current strategies become ineffective means that endolysin research should be an important focus.

## 5. Challenges of Endolysin Development and Engineering 

The potential for endolysins to supplement, or replace antibiotics is exciting. However, this field is still emerging, with very few clinical trials on endolysin-based drugs being conducted. There are a number of challenges and considerations which researchers still face to bring these to market. As highlighted by several studies, the immunogenicity of endolysins must be considered and fully assessed. Undesirable immune responses to these foreign proteins could result in decreased efficacy of the enzymes, or possibly anaphylaxis and autoimmunity [164,165]. While there are studies that have reported on the immunogenicity in the application of endolysins [40,41,42,45,60,63] assessing the degree of immunogenicity in humans using traditional animal models has so far proven unreliable [166]. This was highlighted in studies of SAL200, which showed varying degrees of antibody production between rats, dogs, monkeys and humans [40,41,42]. Although the efficacy of the endolysin may not be observably impacted in vitro or in vivo, the clinical effects may be more significant. Until more human and animal-specific (for animal husbandry applications) clinical trials are conducted, the immunogenic nature of endolysins will remain unpredicatable. 

In light of the current antibiotic resistance crisis, new antibacterials should be rigorously assessed for their potential susceptibility to developed resistance by bacteria. Promisingly, bacterial mutants resistant to endolysins are very infrequent [28,29,30,167]. Fischetti [167] proposed the lack of developed resistance to endolysins has resulted from the evolution of the interactions between bacteriophage and bacteria. The endolysins have evolved to target essential, immutable, molecules within the cell wall, thereby reducing the likelihood of the bacteria developing resistance mechanisms [167]. However, there are also reports of resistance to other peptidoglycan-cleaving enzymes including lysozymes and lysostaphin [168,169,170,171,172,173]. In the event of bacterial adaptation, enzyme engineering may prove useful to combat changes to bacteria in order to maintain the efficacy of endolysins.

The potential for endolysin bioengineering are seemingly endless, including optimizing or changing the catalytic abilities, modifying the lytic spectrum, improving its ability to permeate outer membranes and increasing stability (Table 1). Designing new enzymes requires an understanding of the structure and function of individual domains, the interactions between domains, the placement and composition of linker regions, and elucidation of key residues involved in catalysis. Bioinformatics and structural characterization studies are integral in this process [174]. Often, structural characterization can be achieved by X-ray crystallography, a powerful and effective technique for elucidating high resolution 3-D structures of proteins [11]; however, the limited ability to crystallize endolysins is a major challenge. The majority of endolysin crystal structures published to date are of single domains, with very few full-length endolysin crystal structures having been solved. This is attributed to the short flexible linker regions between domains [8], as protein flexibility is a common hindrance of crystal formation [175]. It is important to study full-length proteins to get a better understanding of the potential synergistic/antagonistic interactions between domains. Because of the difficulties in obtaining endolysin crystals, alternative structural characterization strategies need to be considered. These include the fusion of endolysins to proteins to decrease their flexibility, thereby allowing for crystallography, and other structural elucidation techniques such as nuclear magnetic resonance [176,177] and cryo-electron microscopy [178]. Although these approaches also have limitations, such as physiological relevance or size restrictions, exploration of these techniques may advance the structural characterization of endolysins.

## 6. Conclusions

The field of endolysin research is dynamic, with many potential applications being investigated in the medical, veterinary, and food sectors. The current global crisis of antimicrobial resistance is driving much of this work, with endolysins showing great promise to replace or supplement antibiotics. Engineering endolysins with optimized or new properties provides an opportunity to create even more effective tools. As more bacteriophage endolysins are biochemically and structurally characterized, the ability to design new enzymes improves, therefore expanding the arsenal of lytic tools. However, there are still many challenges that need to be addressed before this technology can be widely adopted by practitioners and industry. While many researchers have described the isolation and in vitro characterization of endolysins, establishing the in vivo efficacy and operating parameters of endolysins for human clinical use, food protection and supplementation, animal husbandry and welfare, and in the environment will be of great importance over the coming years. New technology to cost-effectively scale up endolysin production is also required, as this is currently a significant barrier to implementation. Finally, regulatory pathways need to be established for the use of endolysins in each of the various fields of application, and this can only be achieved by early and effective dialogue with the relevant authorities.

## Figures and Tables

**Table 1:** Possible molecular engineering strategies with potential application(s).

Modification	Property	Example	Reference
Truncation of full-length enzyme	Increased catalytic ability and solubility	CHAPk	Horgan et al. [19]
Fusion of EADs with CBDs of different endolysins	Increased catalytic ability and solubility	ClyS	Daniel et al. [20]
	Increased catalytic ability and broader lytic spectrum	SA2-E-Lyso-SH3b, SA2-E-LysK-SH3b	Schmelcher et al. [21]
	Thermostability	PlyGVE2CpCWB	Swift et al. [22]
Fusion of virion-associated lysin with CBD of endolysin	Increased efficacy	EC300	Proença et al. [23]
Endolysin fusion with OMP	Increased efficacy towards Gram-negative bacteria	OBPgp279, PVP-SE1g-146	Briers et al. [24]
Endolysin fusion with AMP	Increased efficacy towards Gram-negative bacteria	Art-175	Briers et al. [25]
Truncation and site-directed mutagenesis	AMP development	LysAB2	Peng et al. [26]

EAD: enzymatically-active domain; CBD: cell wall-binding domain; OMP: outer-membrane permeabilizer; AMP: antimicrobial peptide.

**Table 2:** Examples of other potential uses of endolysins in foods.

Food	Organism	Endolysin	Reference
Fish	*Shewanella putrefaciens*	ORF62	Han et al. [140]
Vegetable fermentation	*Leuconostoc mesenteroides*	ORF35	Lu et al. [141]
Kimchi	*Lactobacillus plantarum*	SC921 lysin	Yoon et al. [142]
Pears	*Erwinia amylovora*	ΦEa1h lysozyme	Kim et al. [143]
Banana juice	*Salmonella* Typhimurium, *Yersinia enterocolitica*, *Escherichia coli O157:H7*, *Shigella flexneri*	λ lysozyme (with high pressure treatment)	Nakimbugwe et al. [144]
Shellfish	*Vibrio parahaemolyticus*	Lysqdvp001	Wang et al. [145]
Lettuce	*Listeria innocua*	Ply500 (with packaging film)	Solanki et al. [146]
Milk	*Listeria monocytogenes*	PlyP825 (with high pressure treatment)	Misiou et al. [147]
Mozzarella cheese	*L. monocytogenes*	PlyP825 (with high pressure treatment)	Misiou et al. [147]
